# Causal predictive modeling of survival of lung and bronchus cancer patients diagnosed during 2010–2011 in Texas

**DOI:** 10.1371/journal.pone.0333477

**Published:** 2025-10-31

**Authors:** Zeinab Mohamed, Sidketa Fofana, Everado Cobos, Manish K. Tripathi, Tamer Oraby

**Affiliations:** 1 Department of Mathematics, College of Arts and Sciences, Oberlin College, Oberlin, Ohio, United States of America; 2 School of Mathematical and Statistical Sciences, College of Sciences, The University of Texas Rio Grande Valley, Edinburg, Texas, United States of America; 3 Medicine and Oncology, ISU, School of Medicine, University of Texas Rio Grande Valley, McAllen, Texas, United States of America; 4 South Texas Center of Excellence in Cancer Research, School of Medicine, University of Texas Rio Grande Valley, McAllen, Texas, United States of America; Inha University Hospital, KOREA, REPUBLIC OF

## Abstract

**Background:**

Lung and Bronchus cancer is the most fatal type of cancer in the United States. According to the American Cancer Society, there were more than 127,000 deaths from lung cancer in 2023. Lung cancer care cost 23.8 billion dollars in 2020. In Texas, only 22.8% of lung cancer patients survived 5 years or more past diagnosis based on 2012–2018 data.

**Aim:**

This study evaluates the survival length of lung and bronchus cancer patients in Texas using advanced statistical and machine learning methods applied to an 11-year cohort study from Surveillance, Epidemiology, and End Results Program. It also quantifies the causal effect of early (localized) versus late (distant) stage at diagnosis on survival time of those patients. Additionally, it explores the influence of demographic and available clinical factors to assess disparities in survival across different groups.

**Methodology:**

We performed classical survival analyses, followed by causal survival analysis to study the average years lost among different patient groups. Additionally, we performed survival random forest and survival neural network modeling. Finally, we conducted causal inference and causal survival random forest to estimate and predict the average treatment effect of early-stage diagnosis on lung cancer patient survival.

**Results:**

Stage and age are the two most important factors in predicting the survival of patients with lung and bronchus cancer. Lung cancer patients diagnosed with the regional stage have about twice the risk of dying as those in the localized stage at any time, and this risk increases as the stage advances. We also find that the average extended lifetime of the localized stage group was about 4 years compared to survivors diagnosed with the distant stage. It can also extend the probability of survival by up to 50%.

**Conclusion:**

Our study underscores the need for early screening, diagnosis and improving equity in lung cancer patients care, which could lead to improved outcomes and reduced mortality in this high-risk population.

**Impact:**

Understanding lung and bronchus cancer survival using advanced causal inference and predictive modeling techniques, highlights the critical importance of early-stage diagnosis, showing that patients diagnosed at localized stages have a substantially higher survival probability. This research underscores the necessity of promoting early screening and equitable cancer care to improve survival rates and healthcare outcomes for lung and bronchus cancer patients.

## 1. Introduction

According to the American Cancer Society (ACS), lung cancer is by far the leading cause of cancer death in the United States of America, accounting for about 1 in 5 of all cancer deaths. Each year, more people die of lung cancer than of colon, breast, and prostate cancers combined, and the chance that a man will develop lung cancer in his lifetime is about 1 in 16; for a woman, the risk is about 1 in 17 [1].There are 234,580 new cases and 125,070 estimated deaths in 2024, as per the ACS 2024 statistics. Lung and Bronchus cancer represent 11.7% of all new cancer cases in the USA [2]. Furthermore, the estimated national expenditure for lung cancer care in 2020 was 23.8 billion Dollars. The average (per patient) annualized 2007–2013 cancer-attributable costs in 2020 US dollars for medical services related to lung cancer care was $68,293.3 for the initial care of the lung, $12,388.6 for continuing care, and $110,247.8 for the last year of life [3]. Therefore, lung and bronchus cancer are a public health issue and an economic burden for society.

In our study, we focus on the state of Texas since lung cancer is the most lethal cancer in the state as well. In fact, only 22.8% of Texans diagnosed with lung cancer survived for at least 5 years past diagnosis from 2012–2018 data [4]. Furthermore, in Texas, there were 15,533 new lung and bronchus cancer cases in 2020 and 10,792 deaths from the disease in the same year [5].

This research aims to analyze the 11-year cohort study of lung and bronchus cancer survival data in Texas from 2010–2011 [6] to identify the health and socio-economics disparities in lung and bronchus cancer survival that might explain the reasons why some cancer patients live longer than others. We also want to examine the social and environmental effects on the survival of cancer patients due to their protected attributes, such as sex, race, and ethnicity.

Finally, we aim to develop predictive models utilizing causal machine learning and deep learning methods that surpass the correlation relationships between the survival of cancer patients and their features. To address those aims, we will use several statistical and machine learning approaches. In particular, we will use the Kaplan-Meier curve, the log-rank test, Cox proportional hazard regression, matching methods in observational studies, measures of counterfactual fairness, and regression and classification methods of ML/DL, like random forest and neural networks. All those different techniques help better understand the main drivers of lung and bronchus cancer survival, and survivability prediction models from lung cancer can help healthcare professionals and patients effectively handle medical care and costs.

The rest of the paper is organized as follows: a background and literature review in Section II, then a data description and characteristics in Section III, statistical models in Section IV, results in Section V, and finally the discussion and conclusion in Section VI.

## 2. Background and literature review

According to the Centers for Disease Control and Prevention, cancer is a disease in which cells in the body grow out of control. When cancer starts in the lung or/and bronchus, it is called lung and bronchus cancer. There are two major types of lung cancer. Non-small cell lung cancer (NSCLC) accounts for between 80% to 85% of all lung cancer, and small cell lung cancer (SCLC) accounts for 10% to 15% of all lung cancer. In addition, other tumors can occur in the lungs, such as lung carcinoid tumors, adenoid cystic carcinomas, lymphomas, and sarcomas [7]. There can be metastasis to the lung as a secondary organ and from the lung to different organs. Metastatic tropisms refer to the preferences of cancer cells for specific organs or tissues during metastasis. Various factors contribute to these preferences, including the relations between the cancer cells and the microenvironment of the target organ. For instance, breast cancer cells tend to metastasize to the brain, bones, and lungs [8,9], while lung cancer cells often spread to the brain or other lung tissues [10]. Research studies have provided insights into the molecular mechanisms underlying metastatic tropisms and the interactions between circulating tumor cells and the microenvironment of target organs [11–13]. Our study will only focus on cancer, with the primary sites being the lung and the bronchus.

Several risk factors contribute to the development of lung and bronchus cancer. The main risk factor is smoking, and the causative association established of cigarette smoking is lung cancer is well known [14–16], and the length of smoking should be the most vital determinant of lung cancer risk in smokers [17]. About 80% of lung cancer deaths are thought to result from smoking [18]. Therefore, as a society, we need to continue promoting quitting smoking to reduce lung and bronchus cancer considerably. Exposure to carcinogens such as arsenic, nickel, asbestos, silica, and air pollution are important risk factors for lung and bronchus cancer [16,19]. The government and industry should continue to make policies that help protect workers from these exposures, such as avoiding limiting exposure to asbestos in the workplace, particularly in industries like mining. HIV and Chronic pulmonary infections, such as tuberculosis, can lead to lung and bronchus cancer [20,21]. We need to continue monitoring patients with such conditions, and healthy lifestyles, such as eating a lot of fruits and vegetables and limiting the intake of alcohol, can help reduce the risk of getting lung cancer [22,23]. Finally, genetic risk factors like a positive family history of lung cancer are other risk factors for lung and bronchus cancer [24].

Advanced lung and Bronchus cancer can be challenging for survival. Therefore, lung cancer screening is recommended for certain people who smoke or used to smoke, but who do not have any signs or symptoms [25]. The American Cancer Society recommends yearly screening for lung cancer with a low-dose CT (LDCT) scan for people aged 50–80 years who smoke or used to smoke and have at least a 20-pack-year history of smoking. A low-dose CT (LDCT) scan is a screening tool that can help find abnormal lung areas that may be cancer. LDCT helps reduce lung cancer mortality [26]. Therefore, we need to promote early screening worldwide. Since lung and bronchus cancer can be a recurrent or chronic disease, the main goal for survivors is to maximize their survival time. Therefore, it is important to understand why some survivors live longer than others. Early detection and prediction of the depth of survivability from cancer can help both patients and healthcare professionals better manage costs, treatment intensity, and time spent on medical care [27].

The main determinants for lung cancer survival that we will consider in this study are stage, grade, primary site of the cancer, number of cancers each patient has, histology of the cancer, age, race, and income. The literature indicates that the stage at which patients are initially diagnosed is a critical factor in survival [28]. Since we only focus on cancer patients at diagnosis, we will use the localized, regional, distant, and unstaged stages. The National Cancer Institute defined the Localized stage as cancer that is limited to the lung, with no sign that it has spread. The regional stage is cancer that has spread to nearby lymph nodes, tissues, or organs. Distant stage is cancer that has spread to distant parts of the body, such as the brain. An unknown stage occurs when there is insufficient information to determine the stage. According to [29], people diagnosed with small cell lung cancer (SCLC) between 2012 and 2018, the rate of survival by stage after 5 years from diagnosis is the following: 30% for the localized stage, 18% for the regional stage, and then decreases intensely for a distant stage to only 3%. For people diagnosed with non-small cell lung cancer (NSCLC) in the same period, the rate of survival by stage after 5 years from diagnosis is the following: 65% for the localized stage, 37% for the regional stage, and then decreases intensely for a distant stage to only 9%. Therefore, early detection is crucial for survival. A person’s general health tends to diminish as they age. This aging process can also reduce people’s ability to combat diseases such as lung and bronchus cancer [30]. We will check the relevance of aging in our study. Furthermore, racial and sex disparity when it comes to lung cancer survival is well known in oncology [31,32].

Our key motivation for performing this study is to determine the main factors that contribute to patient survival when it comes to lung and bronchus cancer in Texas and to be able to use prediction models to predict patients’ survival function. In the next section, we will focus on data description and summaries.

## 3. Exploratory data analysis

In this retrospective cohort study in Texas, the data for lung and bronchus cancer cases was provided by Surveillance, Epidemiology, and End Results (SEER) Program (www.seer.cancer.gov) SEER*Stat Database: Incidence – SEER Research Plus Limited-Field Data, 22 Registries, Nov 2023 Sub (2000–2020) – Linked to County Attributes – Time Dependent (1990–2022) Income/Rurality, 1969–2022 Counties, National Cancer Institute, DCCPS, Surveillance Research Program, released April 2024, based on the November 2023 submission. In our study, we focus on lung and bronchus cancer patients recorded by the SEER Program in 2010 and 2011 and follow them up to 2021. The study has been determined to be ‘Exempt’ under the Basic HHS Policy for Protection of Human Research Subjects, 45 CFR 46.104(d), with IRB-24–0193 at UTRGV. It has also been deemed exempt from full IRB review at Oberlin College.

The main determinants of lung cancer survival that we will consider in this study are stage, grade, primary site of cancer, the number of cancers each patient has, age, race, treatment that each patient received (chemotherapy, radiation, and surgery), median household income, and the areas where survivors lived. All those variables are considered at diagnosis. Those covariates provided in the SEER data were also chosen based on the literature [28,33–35] and the National Cancer Institute identified those covariates among the important factors in lung cancer survivors [36]. Furthermore, the American Cancer Society agrees that the pathology of lung cancer, such as grade, and stage, helps guide patient care, which has an impact on survival [37].

The Texas Cancer Registry program recorded 18,087 cases of lung and bronchus cancer in white non-Hispanic patients, 2,894 cases of lung and bronchus cancer in black non-Hispanic patients, 2,852 cases in Hispanics, and 473 others. In this data, 45.37% are women.

Table S1 in S1 File shows that at diagnosis, white non-Hispanic patients are much older (68.33% are 65 and older) than Black non-Hispanics (54.8% are 65 and older), than Hispanics (66.02% are 65 and older), and others (58.57% are 65 and older). From this dataset, only 35.25% of lung and Bronchus patients had chemotherapy for treatment.

Based on stage, White non-Hispanics were diagnosed with early-stage (18.79% were diagnosed with localized stage) compared to Black non-Hispanics (15.58% were diagnosed with localized stage), Hispanics (15.71% were diagnosed with localized stage), and others (16.70% were diagnosed with localized stage). Overall, 47.89% of all patients were diagnosed with distant stage, which implies that it is urgent to promote early screening in Texas since we know that advanced lung and Bronchus cancer can negatively impact survival length.

**Fig 1:**
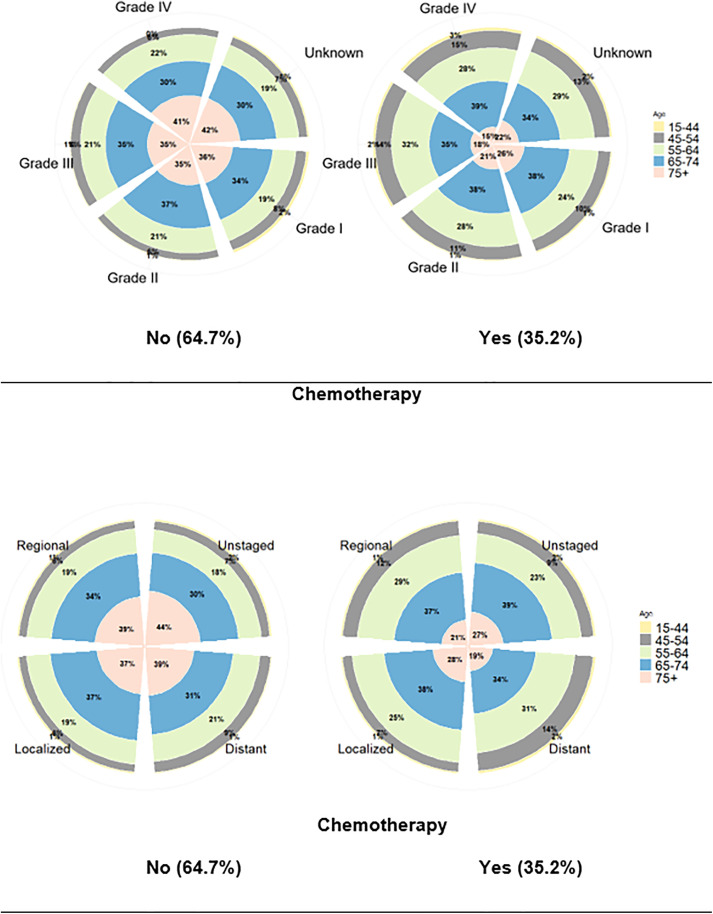
(a) Nightingale rose graph of lung cancer grade by age group and chemotherapy. (b) Nightingale rose graph of lung cancer stages by age group and chemotherapy.

The 15–44 year-old age group is a smaller sector of lung cancer than other age groups in all cases of grades and stages, see Fig 1 (a) and (b). Patients aged 65–74 make up a significant proportion across all stages and grades. Interestingly, the youngest age group (15–44) consistently forms the smallest proportion in all categories, reflecting the lower prevalence of lung cancer in younger individuals. For chemotherapy usage, the percentage of patients receiving chemotherapy is highest in intermediate age groups (55–74). However, older patients (75+) tend to receive chemotherapy less frequently across all stages and grades, with only about 19% of those in the Distant stage and 27% in the unknown stage receiving chemotherapy. This discrepancy in chemotherapy usage suggests that treatment decisions may vary with age and clinical factors, especially in older patients. Generally, there is no striking difference between distributions of grades and stages by age and chemotherapy. However, about 65% of lung cancer patients do not go through chemotherapy.

Furthermore, when it comes to the primary site of lung and bronchus cancer, 45.48% of lung and bronchus cancer start at the upper lobe, lung, 22.18% start at the lung, not otherwise specified (NOS), 22.91% start at the lower lobe, lung and 9.43% start at main bronchus, middle lobe, lung and overlapping lesion of the lung.

Based on the median household income, we can observe that 18.52% of white non-Hispanics were from households with a median income of $75,000.00 or over, compared to 10.57% of Black non-Hispanics, 7.54% of Hispanics, and 19.24% of others. Those income discrepancies may have played a role in the survival length disparities. Finally, 57.53% of the patients live in metropolitan areas with a population of over a million.

## 4. Methods and analyses

### 4.1. Classical and machine learning survival analyses

We perform classical survival analysis methods, encompassing Kaplan-Meier (KM), log-rank test for comparing survival curves, and Cox proportional hazards model for estimating hazard ratios.

A visual representation of an estimate of the survival curve that shows the percentage of observations that have not encountered an event of interest at each time point is the Kaplan-Meier curve. In our study, the event of interest is patients dying from causes that are attributed to lung and bronchus cancer. Additionally, we conducted a log-rank test, also known as the Mantel-Cox test. This nonparametric test is suitable for use in situations where the data are right censored, as in the SEER data. The log-rank test examines whether the survival distributions of two or more groups are the same. Both the KM method and the log-rank test are used to assess the subgroup-specific survival patterns stratified by sex, race, stage, number of tumors, primary site, chemotherapy, grade, systemic therapy, and radiation at diagnosis.

We also examined the assumptions for the Cox PH using Schoenfeld residuals. In case of assumption violation, we will implement a stratified Cox proportional hazards model with stage as the stratification variable, which allows the baseline hazard to vary between the localized and distant stage groups.

We extend the analyses into causal inference; see the next section. We implemented Random Survival Forest (RSF) and Cox Proportional Deep Neural Network (DeepSurv) for advanced predictive modeling. The RSF, an extension of the Random Forests algorithm to survival data, is particularly adept at handling high-dimensional data and accommodating complex interaction effects between covariates without requiring proportional hazards. Key tuning parameters include the number of trees, the depth of the trees, and the number of variables sampled as candidates at each split.

DeepSurv applies deep learning methods to survival analysis, providing a flexible neural network architecture that can capture complex nonlinear relationships between covariates and the risk function. Essential aspects of DeepSurv include the configuration of its neural network layers (such as the number of layers and neurons in each layer), the choice of activation functions, and the optimization algorithm. By leveraging RSF and DeepSurv, we aim to capture a comprehensive understanding of survival data, balancing interpretability with predictive accuracy.

We use the Integrated Brier Score (IBS) and the concordance index (C-index) to assess the performance of the survival analysis methods since the lung cancer data is right censored [38–41]. IBS, which takes values between 0 and 1, is suitable for cases involving censored data and missing information because it utilizes inverse probability censoring weights (IPCW). It gauges the calibration or overall accuracy of the survival probability forecasts made by the model compared to the actual survival outcomes. The C-index statistic also takes values between 0 and 1 and assesses how well the model ranks expected survival times and differentiates patients with different survival histories. An increased C-index value indicates improved discriminating power. Combined small values of IBS and high values of the C-Index, which are always between 0 and 1, mean good predictive survival model performance.

### 4.2. Causal inference and causal machine learning

Causal inference, or inferring the causal impact of one variable on another, is a fundamental problem in observational studies and statistics. The key idea of causal inference is to draw a conclusion based on a set of observed data. Unlike randomized controlled trials, where treatment assignment is independent of any pretreatment variables, observational studies do not enjoy this luxury. The validity of causal inferences from observational studies relies on untestable assumptions. This is particularly important because, in many cases, a wrong conclusion on the cause-and-effect relations can often lead to ineffective or sometimes harmful policies.

The average treatment effects (ATE) play a crucial role in evaluating the efficacy of a treatment or intervention. While this process is relatively straightforward in controlled experiments, it becomes intricate in observational studies where treatment assignment lacks randomization. The complexity arises because treatment exposure may correlate with underlying covariates, which are linked to the potential response variable. As a result, significant imbalances in covariates exist among treatment groups. To ensure a robust causal inference from observational data, employing methods that account for and adjust these confounding effects introduced by background covariates is imperative. Matching techniques have found extensive application in addressing observed covariates when drawing causal inferences from observational data. The comprehensive concept of matching involves any strategy aimed at equalizing or “balancing” the distribution of background covariates between treated and control groups [42]. Essentially, in adjusting data, matching methods work to replicate the conditions of a carefully designed experiment, attempting to create a scenario that could plausibly occur through natural randomization. Mahalanobis distance matching provides an improvement to the nearest neighbors matching. The fundamental problem with basic matching methods is that the distances between the treated and control group members are often unequal in every dimension. When we match individuals based on their characteristics, we look for close matches across all of them.

In this study, we employ four methods for adjusting confounding variables: Propensity Score Matching (PSM), Inverse Probability Weighting (IPW), Mahalanobis Distance Matching with Calipers (MDMC), and Maximum Entropy Weighting (MEW). These methods represent two broad approaches: matching methods and weighting methods. Matching methods create balanced subsets of treatment and control groups by identifying individuals who are comparable. Weighting methods adjust the entire dataset to achieve a covariate balance. Matching methods, such as PSM and MDMC, are well-suited for addressing observed covariate imbalances by explicitly pairing individuals with similar characteristics. Weighting methods, including IPW and MEW, provide a more flexible framework in which a reweighting of the dataset is performed to account for differences in covariates. Together, these two types of methods allow for a robust and comprehensive evaluation of the treatment effect by addressing the influence of confounding through different perspectives. Detailed descriptions of these matching methods, including their mathematical formulations, are provided in S1 Text in S1 File.

We assess the covariate balance and the performance of each of the four methods to identify the most effective method for confounding adjustment in the SEER lung cancer data. In particular, we assess the balance using the standardized mean difference (SMD), plotting the covariate balance before and after adjustment, and carry out a Wilcoxon test.

Furthermore, we conducted a Rosenbaum sensitivity analysis to assess the robustness of the estimated treatment effect to unmeasured confounding [43]. The sensitivity analysis is determined by the parameter Γ, which quantifies the degree to which unmeasured confounding variables can bias the treatment assignment. When Γ = 1, the treatment assignment is inferred to be random—a case mimicking a randomized experiment. For values of Γ larger than 1, individuals with the same observed covariates may differ in their likelihood of receiving treatment due to unmeasured confounders. In that case, one subject may be up to Γ times more likely than another to receive the treatment. In this study, we calculate upper bounds on p-values for a range of Γ values to identify at what value the treatment effect becomes insignificant. If statistical significance disappears for Γ values not much larger than 1, we will infer that the analysis is very sensitive to unmeasured bias. Whereas if significance persists for large Γ values, we deem the results robust to unmeasured confounding.

Causal Random Forest (CRF) is an extension of the traditional random forest algorithm that is designed to estimate treatment effects from observational data, allowing for the identification of causal relationships [44]. CRF is a powerful tool for causal inference in various fields, such as medicine, economics, and social sciences. Unlike traditional random forests, which focus on minimizing prediction error, CRF constructs trees by recursively splitting the data to maximize the heterogeneity of treatment effects within each subset. For each leaf in the tree, the treatment effect is estimated as the difference in average outcomes between treated and control units. These estimates are aggregated across all trees in the forest to provide robust individual treatment effect predictions, taking advantage of out-of-bag (OOB) predictions to avoid overfitting and ensure unbiased estimates.

CRF is particularly useful for identifying and estimating heterogeneous treatment effects, which show how treatment effects vary across different subgroups within the data. It can handle high-dimensional datasets and does not assume a specific functional form for the relationship between covariates and outcomes. Furthermore, using causal survival random forests (CSRF) allows us to estimate the heterogeneous effects of the stage at diagnosis of lung cancer on patients’ survival following the potential outcomes framework of the Rubin Causal Model. CSRFs are extensions of causal random forests that allow handling right-censored survival data [45,46]. The used algorithm also provides measures of variable importance, indicating which covariates are most influential in predicting treatment effects. We used the R package grf (generalized random forests) to implement CRF [47].

## 5. Results

We used Cox Proportional Hazard, classical and causal survival random forests [28], and Neural Networks (NN) to perform our predictive analysis. We will start with the Cox Proportional Hazard model. The Schoenfeld residuals test for the assumption of the Cox Proportional Hazard model indicates that the assumption is violated for the stage variable. To address this, we implemented a stratified Cox proportional hazards model with stage as the stratification variable, which allows the baseline hazard to vary between the localized and distant stage groups. The stratified model yielded results consistent with the original Cox PH model, supporting the robustness of the findings, see S1 Fig in S1 File in the supplementary material. These analyses confirm that the treatment effect is not biased due to the violation of the Cox Proportional Hazard assumption. Furthermore, after fitting the stratified model with stage as the stratification variable, Schoenfeld residual plots indicated no remaining violations of the PH assumption among the other covariates, see S7 Fig in S1 File. Additionally, we examined the interaction of different covariates in the Cox proportional hazards model; however, the results were not statistically significant. Table 1 presents the results for the Cox Proportional Hazard Model. The superscript ** indicates statistical significance at a 5% level (p-values are less than 0.05).

**Table 1:** Statistical inference and hazard ratios for the Cox proportional hazard model. The superscript ** indicates statistical significance at a 5% level (p-values are less than 0.05).

Covariates	Levels	Estimate	Standard Error	Hazard Ratio	95% C.I Hazard Ratio	
					Lower	Upper
**Sex**	Male (reference)					
	Female	−0.18425**	0.01522	0.83173	0.807	0.856
**Race**	White non-Hispanics (reference)					
	Hispanics (all races)	−0.10542**	0.02370	0.89995	0.859	0.942
	Black non- Hispanics	−0.05635**	0.02330	0.94520	0.903	0.989
	Other	−0.43759**	0.05742	0.64559	0.576	0.722
**Age**	Between 15 and 44 (reference)					
	Between 45 and 54	0.46103 **	0.07406	1.58570	1.371	1.833
	Between 55and 64	0.56255**	0.07169	1.75514	1.525	2.019
	between 65 and 74	0.69093**	0.07126	1.99557	1.735	2.294
	75 and above	0.94677**	3.546	2.57738	2.240	2.964
**Chemotherapy**	No/Unknown (reference)					
	Chemotherapy (yes)	−0.19424**	0.01727	0.82346	0.796	0.851
**Grade**	Grade I (reference)					
	Grade II	0.43808**	0.05336	1.54974	1.395	1.720
	Grade III	0.70593**	0.05198	2.02572	1.829	2.243
	Grade IV	0.97106**	0.06639	2.64074	2.318	3.007
	Unknown grade	0.70443**	0.05085	2.02270	1.830	2.234
**Stage**	Localized (reference)					
	Regional	0.70631**	0.02830	2.02651	1.917	2.142
	Distant	1.50078**	0.02671	4.48518	4.256	4.726
	Unknown/ Un-staged	0.71570**	0.03326	2.04562	1.916	2.183
**Primary site of the lung and Bronchus cancer**	Middle lobe, lung (reference)					
	Main Bronchus	0.43135**	0.05219	1.53933	1.389	1.705
	Upper lobe, lung	0.07343	0.04084	1.07619	0.993	1.165
	Lower lobe, lung	0.08588**	0.04238	1.08967	1.0028	1.1840
	Overlapping lesion of lung	0.25793**	0.07469	1.29425	1.118	1.498
	Lung, NOS	0.26586**	0.04263	1.30456	1.200	1.418
**Total in situ\ malignant**	Total in situ\malignant = 1 (reference)					
	Total in situ\malignant = 2	−0.39519**	0.02103	0.67355	0.646	0.701
	Total in situ\malignant ≥3	−0.60294**	0.04155	0.54720	0.504	05936
**Systemic Therapy**	No (reference)					
	Yes	−0.34801**	0.03446	0.70609	0.660	0.755

Compared to males with lung and bronchus cancer, females have a better prognosis with a lower hazard ratio of 0.83173**, see Table 1. Also, in comparison to Whites with lung and bronchus cancer, Blacks present a lower hazard ratio (0.94520**), which means that Whites with lung and bronchus cancer have 5.48% more risk of dying. Furthermore, compared to survivors aged between 15 and 44, survivors 75 and over present a higher hazard ratio (2.57738**). Compared to survivors diagnosed with grade I, survivors with more advanced grades are more likely to die early; for instance, grade IV has a hazard ratio (2.64074**), which means grade IV has 164.07% more risk of dying. Moreover, compared to survivors diagnosed with Localized stage lung cancer, survivors with more advanced stages are more likely to die early; for example, survivors with distant stage have a hazard ratio (4.48518**) that means that at any specific time, survivors diagnosed with distant stage have 348.52% more risk of dying compared to those diagnosed with localized stage. We then need to promote early diagnosis to improve survival length.

Compared to survivors who have one cancer, survivors with multiple cancers are more likely to live longer. For example, survivors with 2 cancers have a hazard ratio (0.67355**), and survivors with three or more have a hazard ratio (0.5472**).

Compared to survivors with the middle lobe of the lung as a primary site, survivors of lung and bronchus cancer that start at the not otherwise specified (NOS) have a hazard ratio of 1.30456**. The ones with the main bronchus as the primary site have a hazard ratio of 1.53933**, the hazard ratio for the lower lobe lung lesion is at 1.08967**, and the hazard ratio for the overlapping lung lesion is at 1.118**. This indicates that survivors with primary sites of NOS lung, main bronchus, lower lobe, and overlapping lesions of the lung are more likely to die early compared to survivors with the middle lobe, lung as the primary site.

Compared to survivors who did not have systemic therapy, survivors who had systemic therapy have a hazard ratio of 0.70609**. This indicates that survivors who did not have systemic therapy are more likely to die early compared to those who have it.

Therefore, based on this Cox regression, the most significant factors are the stage at diagnosis, sex, age, grade, systemic therapy, primary site, the total number of cancers each patient has, and race.

**Fig 2:**
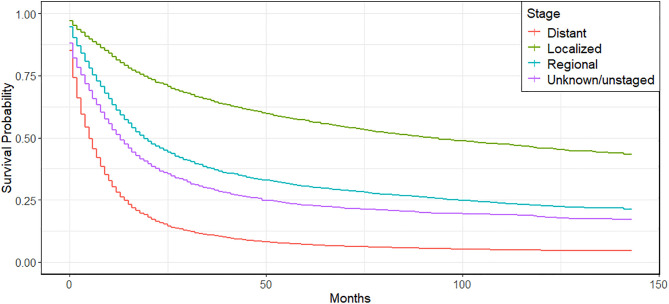
Kaplan-Meier survival curves and log-rank test for lung and bronchus cancer patients based on Stage at diagnosis.

**Fig 3:**
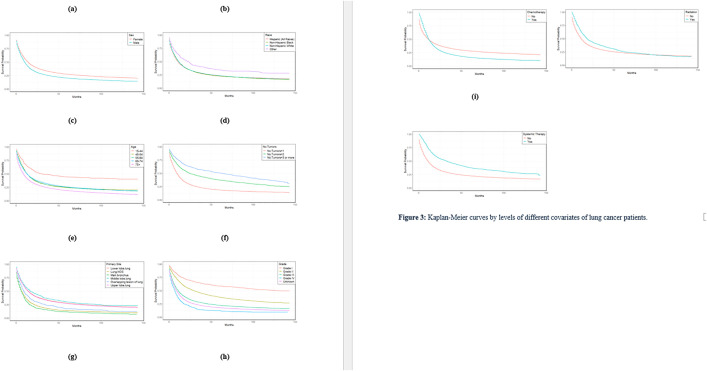
Kaplan-Meier curves by levels of different covariates of lung cancer patients.

Figs 2 and 3 show a relationship between survival probabilities for lung and bronchus cancer patients and the different levels of covariates supported by the statistically significant log-rank test. Fig 2 indicates that the stage at diagnosis plays an important role in determining survival time. For instance, after 60 months (about 5 years), around 62% of patients diagnosed at the localized stage are still alive, while only about 8% of patients with distant-stage lung cancer survive beyond this point. Patients with regional-stage or upstaged cancer show intermediate survival probabilities, with the majority also experiencing a substantial drop in survival within the first 5 years. These results highlight the critical importance of early diagnosis to improve survival outcomes among lung cancer patients, as earlier stages are associated with markedly higher survival rates. From Fig 3(a), it is evident that females have a better survival rate than males for lung and bronchus cancer. Likewise, grade at diagnosis is one of the determinants of survival length for lung and bronchus cancer. Fig 3 (f) shows that after 60 months (5 years), the Grade I (well-differentiated) tumors show the highest survival probability, with approximately 60% of patients surviving. In contrast, Grade IV (undifferentiated) tumors exhibit a much steeper decline, with only around 12.5% of patients surviving after 60 months. Grade II and Grade III show intermediate survival outcomes, with survival probabilities decreasing more gradually than Grade IV but still significantly lower than Grade I. Patients in the unknown grade category follow a similar trend to those with Grade III or IV tumors. Based on the number of cancers each survivor had, see Fig 3 (d), patients with a single tumor have the shortest survival times, with around 81.4% of them having died within 60 months (5 years). In comparison, patients with two tumors fare somewhat better, with approximately 62.7% having died after 60 months. Those diagnosed with three or more tumors exhibit the longest survival, with about 50.5% still surviving beyond the 5-year mark. This type of positive association between survival longevity and the number of tumors should not be misinterpreted. Survival after the diagnosis at the distant stage of lung cancer is generally short enough to allow for the development of further tumors. In the underlying cohort, approximately 81.7% of lung cancer patients who are diagnosed at distant stages died within 24 months, compared with only 30.7% of those diagnosed at localized stages. Moreover, 51.5% of patients with exactly one primary tumor are diagnosed at a distant stage. That proportion falls to 38.4% of patients with two primary tumors, and 28.1% of those with three or more are diagnosed at the distant stage. This pattern reflects survivorship bias, in which patients accumulate additional tumors because they live long enough to develop them and not because multiple cancers induce longer survival. That gives a motivation for performing causal inference.

Regarding the primary sites of lung and bronchus cancer, see Fig 3(e). Survivors whose primary sites are the middle lobe and lung live longer, while those whose primary sites are not otherwise specified have a shorter survival length. Fig 3 (g) for chemotherapy reveals interesting dynamics between short-term and long-term survival. Initially, patients who received chemotherapy had a much higher survival probability, with 97.3% surviving at the time of diagnosis, compared to 85.6% for those who did not receive chemotherapy. However, by 10 months, survival probabilities begin to converge, with 54.3% of chemotherapy patients still alive compared to 51.2% of non-chemotherapy patients. After 20 months, survival probabilities diverge further, with only 33.4% of chemotherapy patients surviving, compared to 40.4% of non-chemotherapy patients. These results indicate that while chemotherapy offers early survival benefits, patients who did not receive chemotherapy show better long-term survival beyond 20 months. This crossing pattern suggests that, although chemotherapy may provide short-term benefits, other factors, such as treatment-related toxicity or disease progression, may ultimately influence long-term outcomes. Finally, based on race and ethnicity groups, black non-Hispanics have a slightly lower survival length compared to other groups, see Fig 3 (b). Therefore, increasing awareness of lung and bronchus cancer within all communities is crucial.

**Fig 4:**
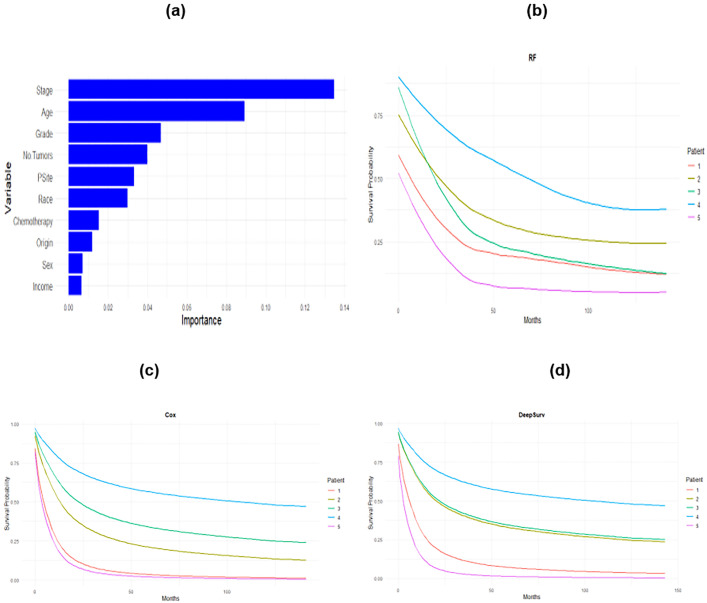
Feature Importance of variables on prediction of the RSF model in (a), predicted survival curves for the first five patients using (b) RF, (c) Cox, and (d) DeepSurv model.

A 5-fold cross-validation was used to identify the final configuration of the best-performing Random Survival Forest (RSF) model, which had a maximum depth of 5, a maximum of 4 features, a minimum of 50 samples per leaf, a minimum sample split of 2, and 1200 trees overall, see S4 Fig in S1 File . Fig 4 (a) shows the importance of the features in RF model. With a C-index of 0.71 and an IBS of 0.078, the RSF model demonstrated its strong calibration and discriminatory skills. Stage and age are the two most important features in predicting the survival of lung cancer patients. The grade and number of tumors are less than one-half of the importance of stage and age.

The DeepSurv model is trained through gradient-based optimization methods, which progressively adjust the neural network weights. This adjustment aims to reduce the Cox partial likelihood loss, thereby enhancing the model’s predictive accuracy. The chosen model configuration features 5 hidden layers with 32, 64, 128, 256, and 512 nodes each, a batch size of 64, dropout of 0.1, and a learning rate of 0.001. The C-index and the IBS for the DeepSurv model are 0.63 and 0.081, respectively. That means that the RSF model outperformed the DeepSurv model. The RSF outperforms both the Cox proportional hazards model and the DeepSurv model, achieving the highest C-index and the lowest IBS, with all differences being statistically significant (paired Wilcoxon signed-rank tests, p-values < 0.05), as shown in S4 Table in S1 File . This confirms that the superior predictive performance of RSF is not only practically meaningful but also statistically robust. The RSF model achieved the lowest prediction error among the Cox and DeepSurv models, S5 Fig in S1 File . Training and validation diagnostics of the DeepSurv model in S6 Fig in S1 File show that training and validation loss decrease consistently, indicating that the model is effectively learning from the training data while also generalizing well to the validation data, as the losses converge and stabilize over time, see S6 Figin S1 File . Fig 4 (b) shows the predicted survival curve for five patients using RF, (c) Cox, and (d) DeepSurv.

To understand the role of stage at diagnosis as the most important variable in prediction, we perform causal inferences for the localized state at diagnosis against the distant stage.

To ensure a robust and comprehensive causal inference from this observational study, we employed and compared four methods for confounding adjustment. We evaluate the efficiency of these four methods in reaching a covariate balance between the treatment and control groups, namely, patients diagnosed in the localized stage and in the distant stage. In that evaluation, we compare the standardized mean differences (SMD) before and after applying the adjustments, see S2 Table and S3 Table in S1 File . Before performing those adjustments, there were large imbalances in the raw data for all covariates where SMD is found to be beyond ±0.1. After applying the adjustment methods, all four methods significantly improved the covariate balance, lowering the SMD value to within ±0.1. MDMC attained an excellent balance in all covariates. At the same time, the PSM achieved a good balance in most covariates but exhibited slightly lower performance for some variables, such as Grade and No. of Tumors. This demonstrates the ability of MDMC to handle high-dimensional covariate space. While IPW had a significant gain in balance for all covariates, MEW achieved the most precise balance among all methods. The covariate balance plot, see S2 and S3 Figs in S1 File in the supplementary material, provides a graphical representation of the degree of reduction in imbalance for each method relative to the unadjusted data.

After confirming the successful matching, we calculated the average treatment effect (ATE). ATE provides an estimate of the causal impact of being diagnosed with localized versus distant lung cancer type. We compared ATE estimates across the four confounding adjustment methods. The results show that Mahalanobis Distance Matching with Calipers (MDMC) produced the largest reduction in the likelihood of death (ATE = ‒0.37) with the smallest standard error (0.0061), indicating high precision while supported by the effective adjustment for confounding. Maximum Entropy Weighting (MEW) also showed a substantial effect (ATE = ‒0.36) with high precision (standard error = 0.0070). This highlights its capacity to balance covariates efficiently across the treatment and control groups by weighting the subjects, while maintaining comparable precision to matching methods like MDMC. Propensity Score Matching (PSM) resulted in an ATE of ‒0.31 (standard error = 0.0093), which reflects its ability to create a balanced matched dataset with similar ATE but slightly lower precision, though it relies on a subset of the data. Inverse Probability Weighting (IPW) produced an ATE of ‒0.29 with a standard error of 0.0098. While IPW retains the full dataset, it slightly reduced the effect size compared to the other methods. The consistency in the direction and significance of the ATE across these two types of matching and weighting methods underscores the robustness of our findings to methodological choices. Each of these methods offers unique strengths and trade-offs, including differences in subjects’ retention, precision of estimates, and covariate balance.

The results of Rosenbaum’s sensitivity analysis were that the treatment effect was still statistically significant (p < 0.05) for values of the parameter Γ as high as 1.5, and this implies that any hidden confounding variables would need to raise the odds of treatment assignment by at least 50% to violate our findings. This is such that our results exhibit sensitivity to moderate levels of hidden bias. Additionally, the covariate balance plot showed substantial improvement in balance for all methods, see S3 Fig in S1 File in the supplementary material.

**Fig 5:**
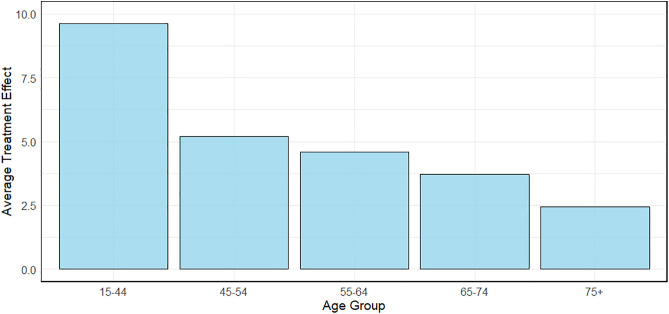
Average treatment effect of localized versus distant stages at diagnosis (in years) by age group.

In addition to calculating the ATE, a significant difference was found between the two groups using the Wilcoxon test with a p-value (<2.2e-16). Patients diagnosed at a localized stage had an average extended survival time of about 4 years compared to those with a distant stage. Also, a relative risk of 1.5 shows that patients with distant stages have 50% more risk of dying than patients with localized stages. Notably, the ATE decreased with age, see Fig 5, showing a nearly double effect size on survival for patients under 40 compared to those aged 40–49, with the effect diminishing gradually in older age groups. The results from the stratified Cox proportional hazards model corroborate these findings. The model revealed that patients diagnosed with distant stage had a hazard ratio of 4.99 (95% CI: 4.64–5.37), indicating a significantly higher risk of mortality compared to those diagnosed at a localized stage. The high significance of this result (p < 2e-16) and the model’s concordance of 0.73 support the robustness of this outcome. This reinforces the importance of early diagnosis and treatment to improve survival outcomes in lung cancer patients.

**Fig 6:**
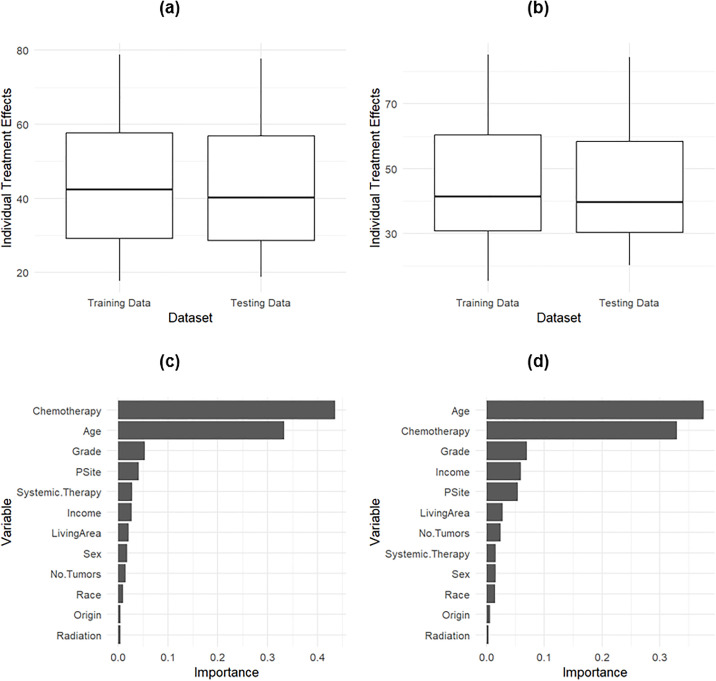
Predicted individual treatment effect of detection at the Localized Stage versus at the Distant stage at a 120-month horizon in (a) and a 140-month horizon in (b). The training of the data and the predictions are performed using causal survival random forest (CSRF) trained at each horizon separately. The variable importance of the 120-month horizon is in **(c)**, and the 140-month horizon is in **(d)**.

We also used a causal survival random forest (CSRF) to calculate the average treatment effect (ATE), which, in this case, represents the difference in the survival probabilities between patients diagnosed at the localized stage and those diagnosed at the distant stage. There is an increase in survival length due to localized stage diagnosis by an average of 45 months, with a standard error of 0.12 months, over a 120-month horizon, as shown in Fig 6 (a). The increase is by an average of 45 months and a standard error of 0.13 months in a 140-month horizon, see Fig 6 (b). Testing data confirm the estimates for both horizons in Figs 6(a) and 6 (b). For the 120-month horizon, the most important variables in the prediction, in order, are: undergoing chemotherapy, patient age, and the grade level of the lung cancer, as shown in Fig 6 (c). The first two variables change order for the 140-month horizon, see Fig 6 (d).

## 6. Discussion and conclusions

In this study, we employ various advanced statistical and machine learning methods to predict and understand the survival of lung and bronchus cancer patients diagnosed in Texas from 2010 to 2011. The main finding of the study emphasizes the importance of the early diagnosis of lung cancer and the important roles of other demographic and clinical factors on the survival outcomes of lung cancer patients.

The most critical finding of this study is the important impact of the stage at diagnosis on the survival of lung cancer patients. The study’s results show that lung cancer patients diagnosed at a localized stage have substantially longer survival periods than those diagnosed at later stages. For instance, patients with localized stage cancer had an average of 45 months longer survival period over a 140-month horizon than those diagnosed at the distant stages. This highlights the need to promote early screening of lung cancer and diagnosis to improve patient survival.

Our study also identifies the sex and racial disparities in survival outcomes of lung cancer patients. In particular, female patients of lung and bronchus cancer have a better prognosis compared to male patients. Moreover, Hispanic patients have a higher hazard ratio than other races, indicating a greater mortality risk when compared to their White non-Hispanic counterparts. That finding underscores the necessity for targeted interventions and resource allocation to address those demographic disparities to improve equity in cancer care.

The survival advantage of patients with multiple tumors that we found in this study, using statistical non-causal methods, is most likely due to the survivorship bias. When patients survive long enough after their initial diagnosis, they can develop or be diagnosed with additional tumors, whereas patients with more aggressive disease may die earlier before developing further tumors. As a result, the multiple-tumor group of patients will counterintuitively show longer survival times. Using causal methods showed that the number of tumors is not as important a predictor of survival as it appears in the non-causal methods.

Besides classical survival analyses that we conducted in this study, we performed stratified Cox Proportional hazard analyses. Although the Cox Proportional hazard assumption was violated for the Stage variable, i.e., the effect of cancer stage on survival was not proportional over time, the stratified Cox proportional hazards model, stratifying by Stage, yielded results consistent with the original Cox model. Furthermore, the stratified model produced stable estimates, which agreed with the survival pattern as revealed in the Kaplan-Meier curves. We also compared the performance of different predictive models, including Cox proportional hazard, random survival forest (RSF), and neural networks of DeepSurv models. The RSF model demonstrates the best performance with the highest C-index and lowest Integrated Brier Score [44], indicating its superior predictive accuracy and reliability.

The superior performance demonstrated by the survival random forest (SRF) over Cox proportional hazard (CPH) and Deepsurv is an important conclusion in our study. While Deepsurv can handle nonlinearities in the survival relationship, like CPH, it assumes a proportional hazard relationship. Meanwhile, SRF does not require the proportional hazard assumption; additionally, it can handle censoring, non-additivity, and nonlinearities in the survival relationship studied using high-dimensional data. Unlike Deepsurv, SRF is moderately interpretable, with feature importance measures, and can be used for small sample sizes, making it competitive with CPH. While other methods can handle nonlinearities in survival relationships like P-splines, they do not handle censoring and non-proportional hazards by default, and they are computationally intensive, especially with high-dimensional data.

According to our findings in this paper, it is essential to investigate whether the survival differences are causally attributed to the stage of diagnosis and not confounded by other variables, such as demographic and/or clinical covariates. To address this problem, we employed causal inference with the treatment variable defined as Stage (1 = localized, 0 = distant). We applied four methods for confounding adjustment—Propensity Score Matching (PSM), Mahalanobis Distance Matching with Calipers (MDMC), Inverse Probability Weighting (IPW), and Maximum Entropy Weighting (MEW). We demonstrated their varied performance in eliminating covariate imbalance and estimating the average treatment effect (ATE). Each approach has some unique advantages and disadvantages that need to be carefully analyzed in deciding the most appropriate approach for a specific research study. While the MEW performed the best in terms of achieving covariate balance and showed outstanding performance on all the covariates, the ATE for MDMC was ‒0.37 with the smallest standard error (0.0061) among all other methods. This result indicates that cancer patients diagnosed at a localized stage had an average extended survival of about 4 years compared to their matches who were diagnosed at the distant stage. Moreover, a relative risk of 1.5 indicates that lung cancer patients who are diagnosed at the distant stages have 50% more risk of dying than patients diagnosed at the localized stages.

To assess the effect of residual and unmeasured confounding on the findings of this study, we conducted a Rosenbaum sensitivity analysis. The analyses confirmed the robustness of the results of the causal methods to moderate levels of unmeasured confounding.

Our study also uses advanced causal inference methods, such as Causal Survival Random Forest, a type of Causal Machine Learning model, which allows for a more accurate estimation of the effects of early-stage diagnosis and survival probabilities. These methods address the limitations of traditional survival analysis and machine learning by considering the underlying causal relationships between variables and matching subjects using confounders like race and age.

The findings from our study provide valuable insights into healthcare policy and practices. To improve lung and bronchus cancer survival rates, it is crucial to promote early screening and diagnosis, especially in high-risk and underserved populations. We also need to address sex and racial disparities through targeted healthcare initiatives. There is a general need to enhance access to quality care for lower-income and rural populations and develop comprehensive care strategies for patients with multiple cancers.

In future work, we will explore the underlying causes of sex and racial disparities in lung cancer survival. We will also consider the long-term outcomes of patients with multiple cancers and their quality of life. Moreover, we will study targeted interventions, including healthcare access for underserved populations, which might assist in improving early diagnosis and better survival outcomes.

Additional factors, such as a patient’s comorbidities and health-related behaviors like drinking or smoking, may affect the survivability of lung and bronchus cancer. A missing treatment regimen is also another limitation in this study. Nevertheless, the SEER data used here do not offer those details about lung cancer patients. However, when we performed the Rosenbaum sensitivity analysis, it showed that the results were moderately robust to unmeasured confounding. In the future, we will utilize electronic health records and external data to analyze and validate the results presented in this paper.

By using advanced causal predictive modeling, our study provides a novel and robust framework for predicting the survival outcomes of lung and bronchus cancer patients. The insights gained can guide more effective healthcare policies and interventions, ultimately leading to better patient care and outcomes.

## Supporting information

S1 File **S1 Table.** Summaries of lung cancer patients diagnosed during 2010–2011 in Texas. **S1 Fig.** Hazard ratios of the original and stratified, by Stage, Cox Proportional Hazard. **S2 Fig.** Counts distribution for Covariates before (left panels) and after (right panels) Mahalanobis distance matching to Study Stage of cancer at diagnosis. **S3 Fig.** Covariate balance plot. The plot represents the standardized mean differences (SMD) before and after applying the four methods for confounding variable adjustment: Propensity Score Matching (PSM), Inverse Probability Weighting (IPW), Mahalanobis Distance Matching with Calipers (MDMC), and Maximum Entropy Weighting (MEW). **S2 Table.** Standardized mean difference (SMD) of all covariates before and after matching with their p-values. A nonsignificant statistical test indicates successful matching.**S3 Table.** Balanced numbers in both groups of treatment after matching. **S4 Fig.** Error rate and number of trees in prediction of RSF model. **S5 Figure.** Prediction error for Cox, RSF and DeepSurv. **S6 Figure.** Training and validation loss of the DeepSurv. **S7 Figure.** Schoenfeld residual plots. **S4 Table.** The C-index and the IBS statistics for the three models: Cox Proportional Hazard, Random Survival Forest (RSF), and Cox Proportional Deep Neural Network (DeepSurv). **S1 Text**. Matching Methods.
(ZIP)
