# Supplementary material for: Causal predictive modeling of survival of lung and bronchus cancer patients diagnosed during 2010–2011 in Texas
Source: PLoS One. 2025 Oct 31;20(10):e0333477. doi: 10.1371/journal.pone.0333477 (PMC12578144; doi:10.1371/journal.pone.0333477)
Supplement: S1 File — S1 Table. Summaries of lung cancer patients diagnosed during 2010–2011 in Texas. S1 Fig. Hazard ratios of the original and stratified, by Stage, Cox Proportional Hazard. S2 Fig. Counts distribution for Covariates before (left panels) and after (right panels) Mahalanobis distance matching to Study Stage of cancer at diagnosis. S3 Fig. Covariate balance plot. The plot represents the standardized mean differences (SMD) before and after applying the four methods for confounding variable adjustment: Propensity Score Matching (PSM), Inverse Probability Weighting (IPW), Mahalanobis Distance Matching with Calipers (MDMC), and Maximum Entropy Weighting (MEW). S2 Table. Standardized mean difference (SMD) of all covariates before and after matching with their p-values. A nonsignificant statistical test indicates successful matching.S3 Table. Balanced numbers in both groups of treatment after matching. S4 Fig. Error rate and number of trees in prediction of RSF model. S5 Figure. Prediction error for Cox, RSF and DeepSurv. S6 Figure. Training and validation loss of the DeepSurv. S7 Figure. Schoenfeld residual plots. S4 Table. The C-index and the IBS statistics for the three models: Cox Proportional Hazard, Random Survival Forest (RSF), and Cox Proportional Deep Neural Network (DeepSurv). S1 Text. Matching Methods. (ZIP) [file pone.0333477.s001.zip › Supporting information/S1TEXT.docx]

S1 text. Matching Methods

Propensity Score Matching (PSM) estimates the probability of receiving the treatment, called propensity score, based on covariates. Then, subjects in the treatment and control groups with similar propensity scores are matched. PSM simplifies the multidimensional balancing problem into a single score, but it depends heavily on specifying the correct propensity score model [1]. Mahalanobis Distance Matching (MDM) uses the Mahalanobis distance to match subjects based on the multivariate structure of the covariates. MDMC ensures that matches are as close as possible across all covariates, which provides a robust approach to balancing treatment and control groups. When combined with calipers based on the propensity scores, MDMC performs better in a way that restricts matching to subjects with similar propensity scores to prevent inadequate matches [2].

Inverse Probability Weighting (IPW) assigns weights to treated and control subjects. Treated subjects are weighed by the reciprocal of the propensity score and control subjects are weighed by the reciprocal of one less the propensity score. IPW balances the covariates between the two groups as it creates a pseudo-population in which the treatment assignment and covariates are independent [3].

Maximum Entropy Weighting (MEW) adjusts the weights of the subjects to achieve covariate balance while maximizing entropy. MEW ensures that the weights remain as close as possible to be uniform to preserve the control group representation. For categorical covariates, MEW balances the proportions of each category to guarantee that the weighted distribution of the control group matches that of the treatment group for each covariate’s categories [4].

Details about Mahalanobis matching method, including mathematical formulations, are provided in next.

Let $\mathbf{X}$ denotes the vector of covariates for a particular patient and let $Z$ be the binary treatment indicator, (Z = 0 for distant stage, Z = 1 for localized stage). The propensity score, $e\left( \mathbf{X} \right)$, is the probability of being assigned to the treatment group (being diagnosed at the localized stage) given the observed covariates, $\mathbf{X}$

$e\left( \mathbf{X} \right) = P\left( Z = 1 | \mathbf{X} \right)$

Patients who are diagnosed at the distant and localized stage selected to have the same value of $e\left( \mathbf{X} \right)$ will have the same distributions of $\mathbf{X}$. Exact matching on $e\left( \mathbf{X} \right)$ will tend to balance the $\mathbf{X}$ distributions in the distant and localized stage groups.

In practice, $e\left( \mathbf{X} \right)$ is estimated using logit model (Cox 1970):

$q(\mathbf{X})\equiv$logit$\left[ e\left( \mathbf{X} \right) \right]=\beta_{0} + \beta_{1}X_{1} + \beta_{2}X_{2} + ... + \beta_{p}X_{p}.$

The goal is to balance the distribution of covariates between the groups so that:
 Covariate balance: $\mathbf{X}_{\{Z=1\}} \approx\mathbf{X}_{\left\{ Z=0 \right\}}\boldsymbol{.}$
Mahalanobis metric matching, initially introduced by Cochran and Rubin (1973) and further developed by Rubin (1976a), has been extensively studied by Carpenter (1977) and Rubin (1979, 1980). This method involves arranging the treated (being diagnosed at the localized stage) and control groups (being diagnosed at the distant stage) in a random order. Matching begins by pairing the first treated individual with the closest control individual of the same sex, for example, with the "closeness" between them determined using the Mahalanobis distance as follows:

$M(\mathbf{X}_{i},\mathbf{X}_{j}) ={(\mathbf{X}_{i}-\mathbf{X}_{j})}^{'}\sum^{-1}(\mathbf{X}_{i}-\mathbf{X}_{j})$

where Σ is the covariance matrix of the covariates $\mathbf{X}$ and $(\mathbf{X}_{i}-\mathbf{X}_{j})$is the difference between the covariate vectors of patients i and j.
Mahalanobis distance ensures that covariates are balanced not only individually but also in terms of their relationships (e.g., interactions and correlations). To further improve matching quality, this method incorporates propensity score calipers, Althauser and Rubin 1971). A caliper restricts matches patients whose propensity scores $e\left( \mathbf{X} \right)$ differ by no more than a pre-specified threshold δ, i.e. $\left| e\left( \mathbf{X}_{i} \right)-e\left( \mathbf{X}_{j} \right) \right|\leq\delta$. This ensures that matches are similar in both their overall covariates (via Mahalanobis distance) and their likelihood of treatment assignment (via propensity score).

Algorithm for Mahalanobis distance matching within propensity score calipers, Rosenbaum, P. R., & Rubin, D. B. (1985):

1. **Randomize the Treated Group**: Patients diagnosed in the localized stage (treated group) are randomly ordered to ensure unbiased selection during the matching process.
2. **Apply Caliper Matching on Propensity Scores**: For the first localized stage patient, identify all distant stage patients whose $\left| e\left( \mathbf{X}_{i} \right)-e\left( \mathbf{X}_{j} \right) \right|\leq\delta$. This ensures that only distant stage patients with similar likelihoods of being treated are considered.
3. **Perform Nearest Mahalanobis Distance Matching Within Calipers**: From the subset of distant stage patients defined in step 2, select the one who is most similar to the localized stage patient in terms of their covariates $\mathbf{X}$, as determined by the Mahalanobis distance. This ensures that the matched patients are closely aligned not only on propensity score but also on their other characteristics.
4. **Iterate and Update**: Once a match is made, remove both the matched localized stage patient and distant stage patient from their respective lists. Repeat the process for the next localized stage patient, starting again from step 2.

This combination achieves a balance of both global structure (via propensity scores) and local structure (via Mahalanobis distance). Rubin (1979) demonstrated that this method effectively balances not only the individual covariates but also their interactions and higher-order terms.

**References**

1. Rubin, D.B., *Bias Reduction Using Mahalanobis-Metric Matching.* Biometrics, 1980. **Vol. 36, No. 2**: p. 293-298.

2. Rubin, P.R.R.a.D.B., *Constructing a Control Group Using Multivariate Matched Sampling Methods That Incorporate the Propensity Score.* The American Statistician, 1985. **39**: p. 33-38.

3. Austin, P.C. and E.A. Stuart, *Moving towards best practice when using inverse probability of treatment weighting (IPTW) using the propensity score to estimate causal treatment effects in observational studies.* Stat Med, 2015. **34**(28): p. 3661-79.

4. Hainmueller, J., *Entropy Balancing for Causal Effects: A Multivariate Reweighting Method to Produce Balanced Samples in Observational Studies.* Political Analysis 2012. **Vol. 20, No. 1**(WINTER 2012): p. pp. 25-46
