# Supplementary material for: Causal predictive modeling of survival of lung and bronchus cancer patients diagnosed during 2010–2011 in Texas
Source: PLoS One. 2025 Oct 31;20(10):e0333477. doi: 10.1371/journal.pone.0333477 (PMC12578144; doi:10.1371/journal.pone.0333477)
Supplement: S1 File — S1 Table. Summaries of lung cancer patients diagnosed during 2010–2011 in Texas. S1 Fig. Hazard ratios of the original and stratified, by Stage, Cox Proportional Hazard. S2 Fig. Counts distribution for Covariates before (left panels) and after (right panels) Mahalanobis distance matching to Study Stage of cancer at diagnosis. S3 Fig. Covariate balance plot. The plot represents the standardized mean differences (SMD) before and after applying the four methods for confounding variable adjustment: Propensity Score Matching (PSM), Inverse Probability Weighting (IPW), Mahalanobis Distance Matching with Calipers (MDMC), and Maximum Entropy Weighting (MEW). S2 Table. Standardized mean difference (SMD) of all covariates before and after matching with their p-values. A nonsignificant statistical test indicates successful matching.S3 Table. Balanced numbers in both groups of treatment after matching. S4 Fig. Error rate and number of trees in prediction of RSF model. S5 Figure. Prediction error for Cox, RSF and DeepSurv. S6 Figure. Training and validation loss of the DeepSurv. S7 Figure. Schoenfeld residual plots. S4 Table. The C-index and the IBS statistics for the three models: Cox Proportional Hazard, Random Survival Forest (RSF), and Cox Proportional Deep Neural Network (DeepSurv). S1 Text. Matching Methods. (ZIP) [file pone.0333477.s001.zip › Supporting information/S2 Figure.docx]

| Before | After |
| --- | --- |
| 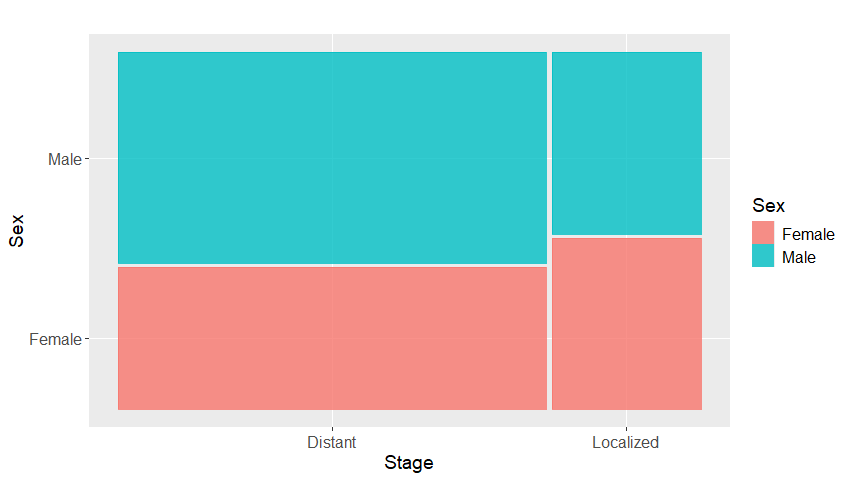 | 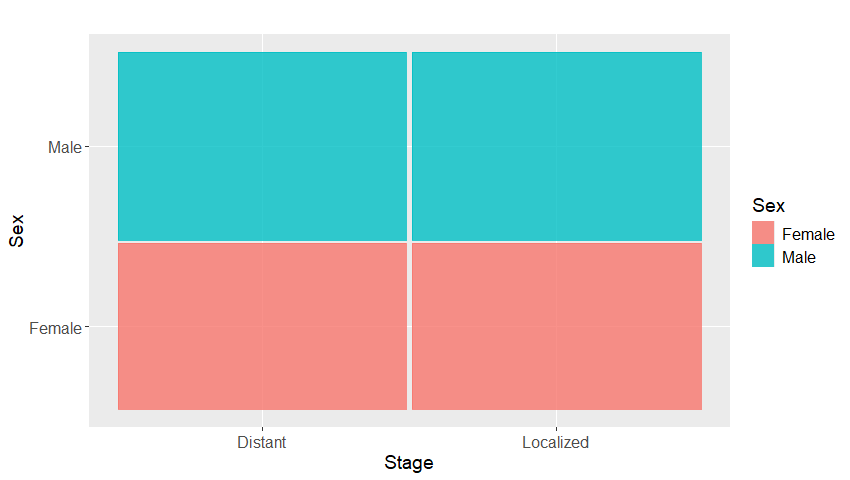 |
| Before | After |
| 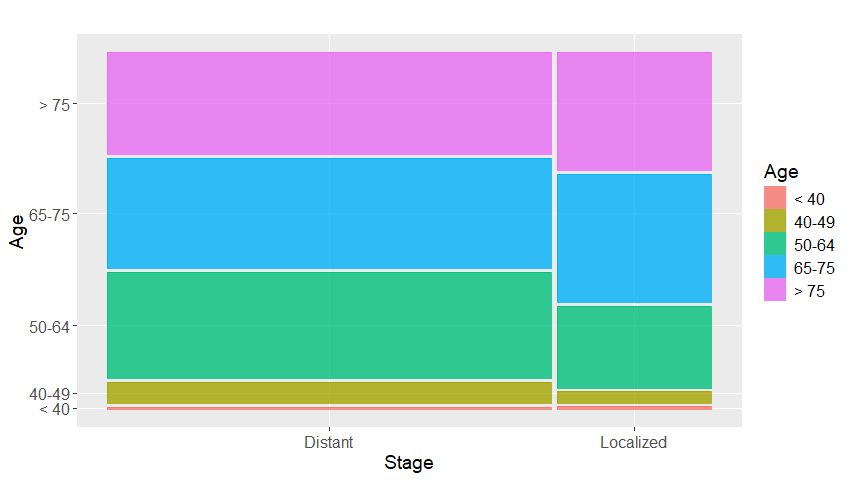 | 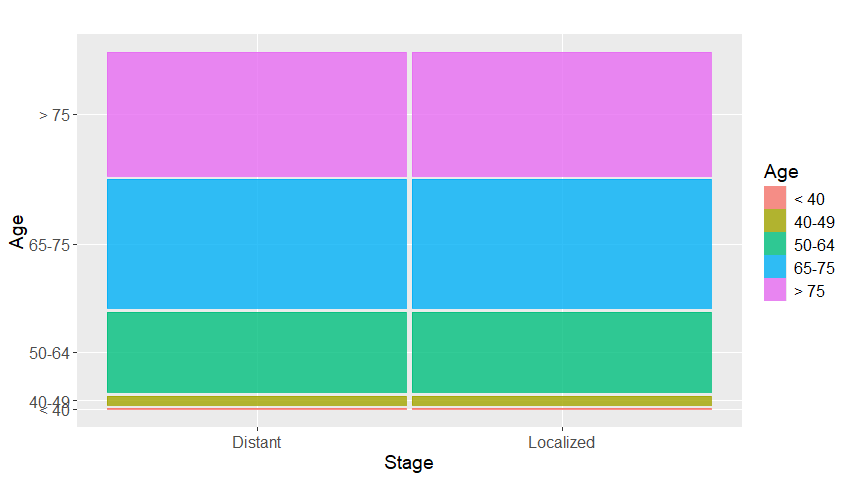 |
| Before | After |
| 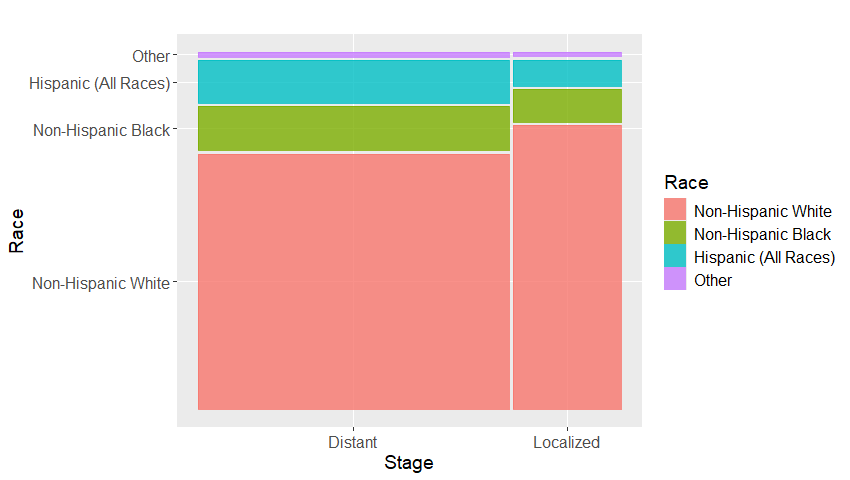 | 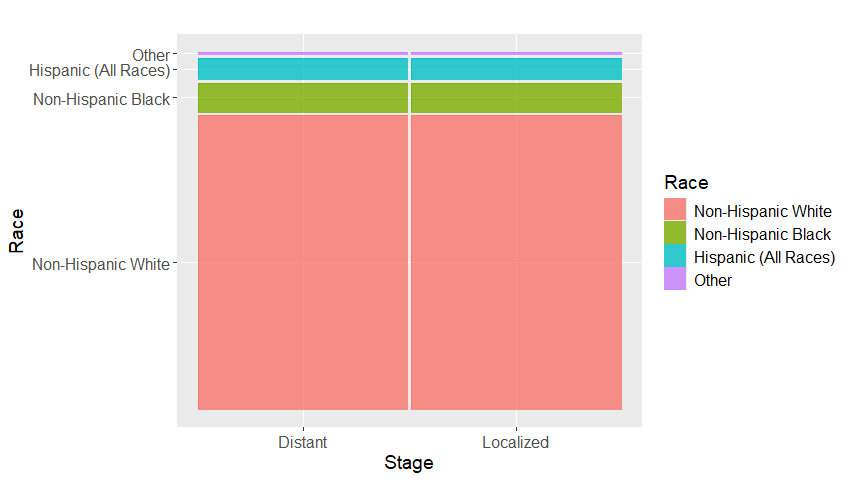 |
| Before | After |
| 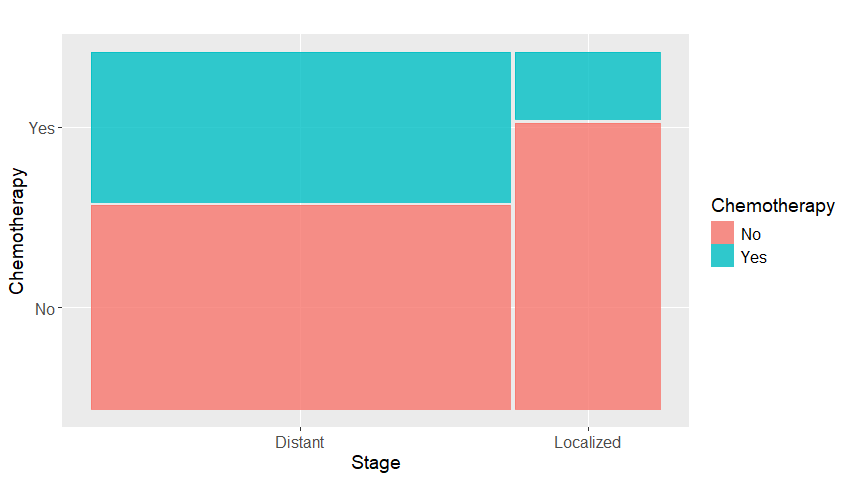 | 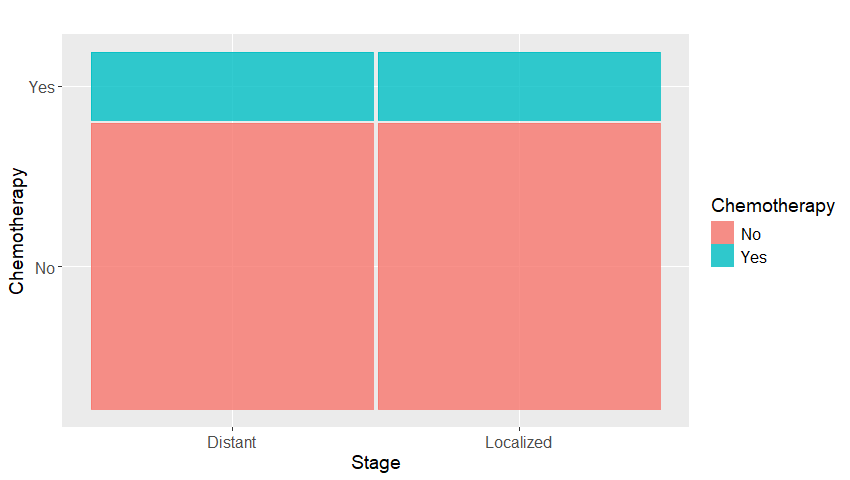 |
| Before | After |
| 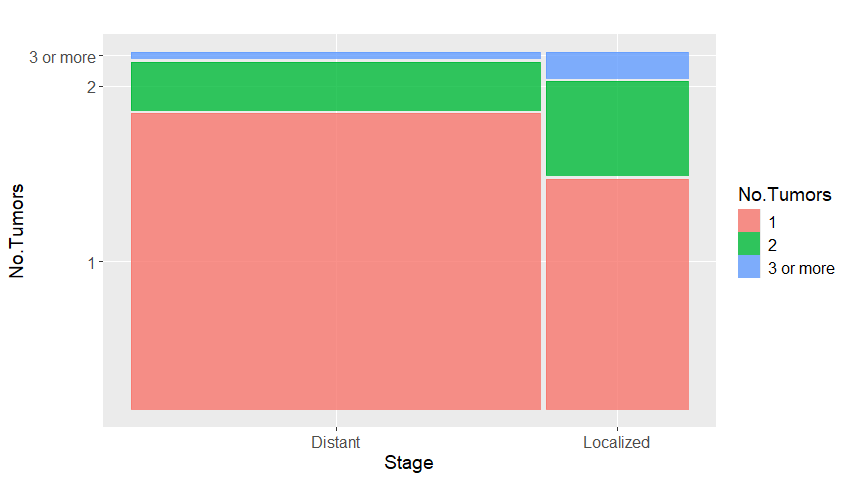 | 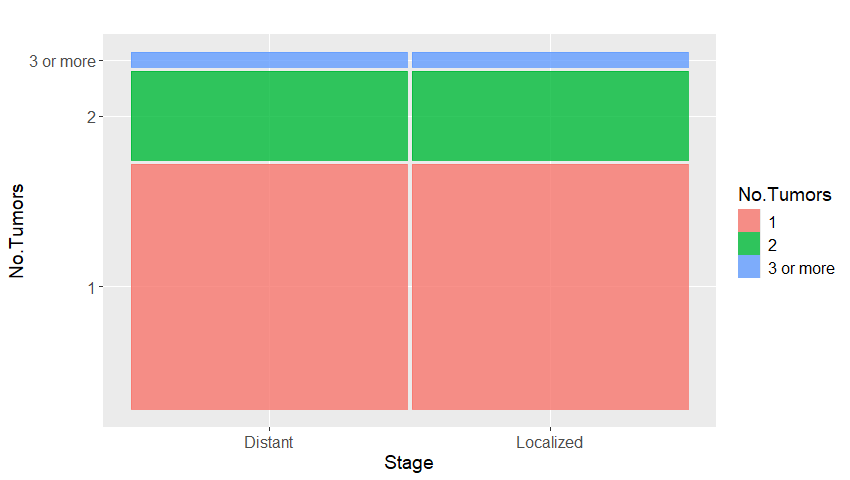 |
| Before | After |
| 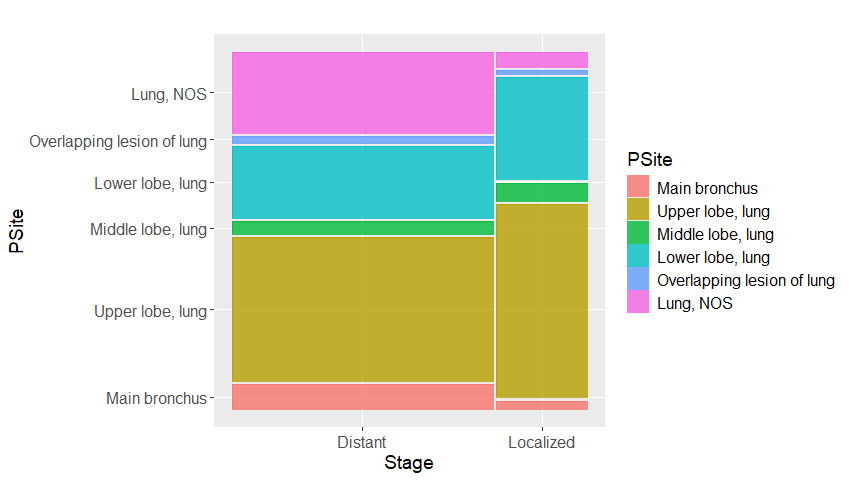 | 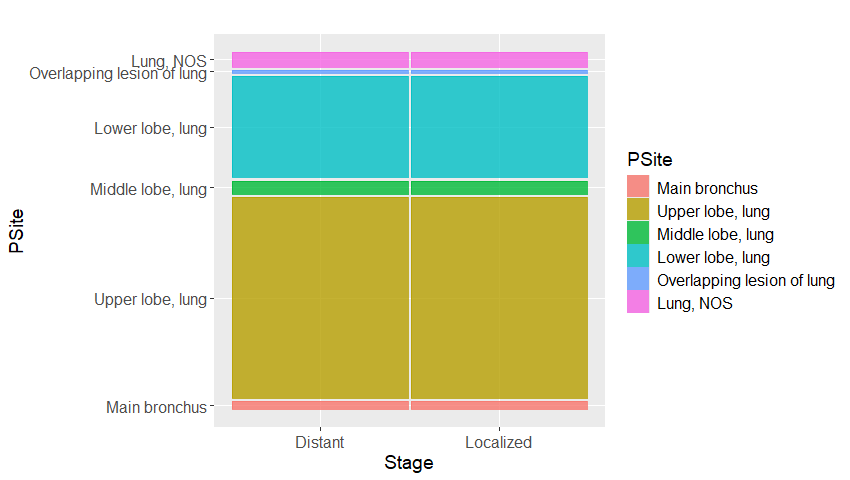 |
| Before | After |
| 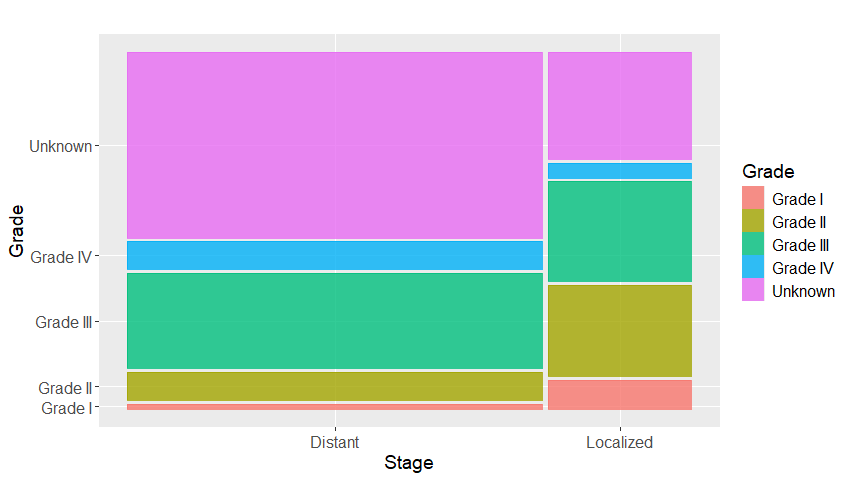 | 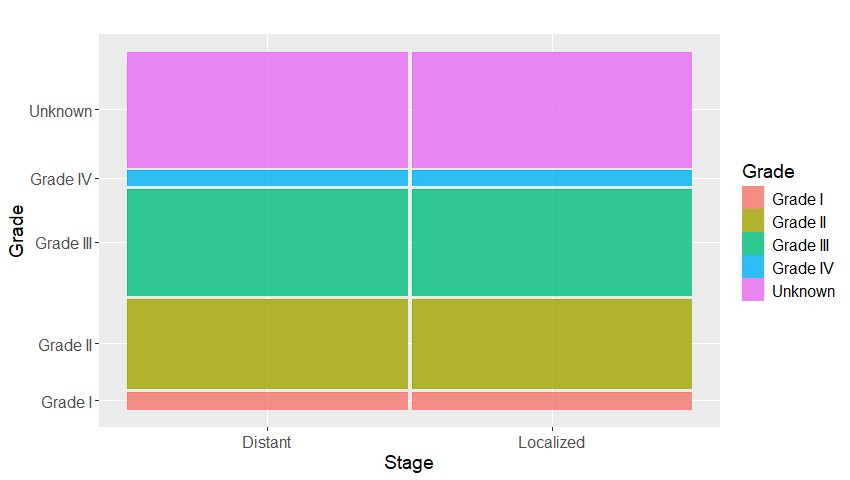 |

**S2 Figure:** Counts distribution for Covariates before (left panels) and after (right panels) Mahalanobis distance matching to Study Stage of cancer at diagnosis.
